# Hematohidrosis: A Rare Case of Bleeding Ears

**DOI:** 10.7759/cureus.63113

**Published:** 2024-06-25

**Authors:** Abrar Nasir, Ng Chong Sian, Asma Abdullah

**Affiliations:** 1 Head and Neck Surgery, Department of Otorhinolaryngology-Head and Neck Surgery, Faculty of Medicine, Universiti Kebangsaan Malaysia, Kuala Lumpur, MYS; 2 Otorhinolaryngology, Hospital Tengku Ampuan Rahimah, Klang, MYS; 3 Otolaryngology, Universiti Kebangsaan Malaysia Medical Centre, Kuala Lumpur, MYS

**Keywords:** self-limiting, bleeding ear, oral propranolol, external ear, hematohidrosis

## Abstract

Hematohidrosis is a rare disease and should be treated as a diagnosis of exclusion. Its pathogenesis remains unclear, though several theories have been proposed. It is often associated with psychological stress and anxiety, with the most commonly affected areas being the ears, nose, and oral mucosa. There is no specific management modality for hematohidrosis yet, as it is generally self-limiting. Enhanced monitoring and surveillance are required to avoid complications and determine the severity of the condition.

## Introduction

Hematohidrosis is a rare condition characterized by humans excreting blood from sweat glands. It is aggravated by psychological stress and has an unknown exact pathogenesis [1]. Throughout history, sweating blood was described to have spiritual connotations in religious scriptures, and Aristotle reported it as early as the third century BCE [2]. Based on a few reported cases in the literature, this condition predominantly affects young females [3,4]. In this report, we discuss a case of hematohidrosis in a female patient and describe the treatment and subsequent follow-up progress.

## Case presentation

**Figure 1:**
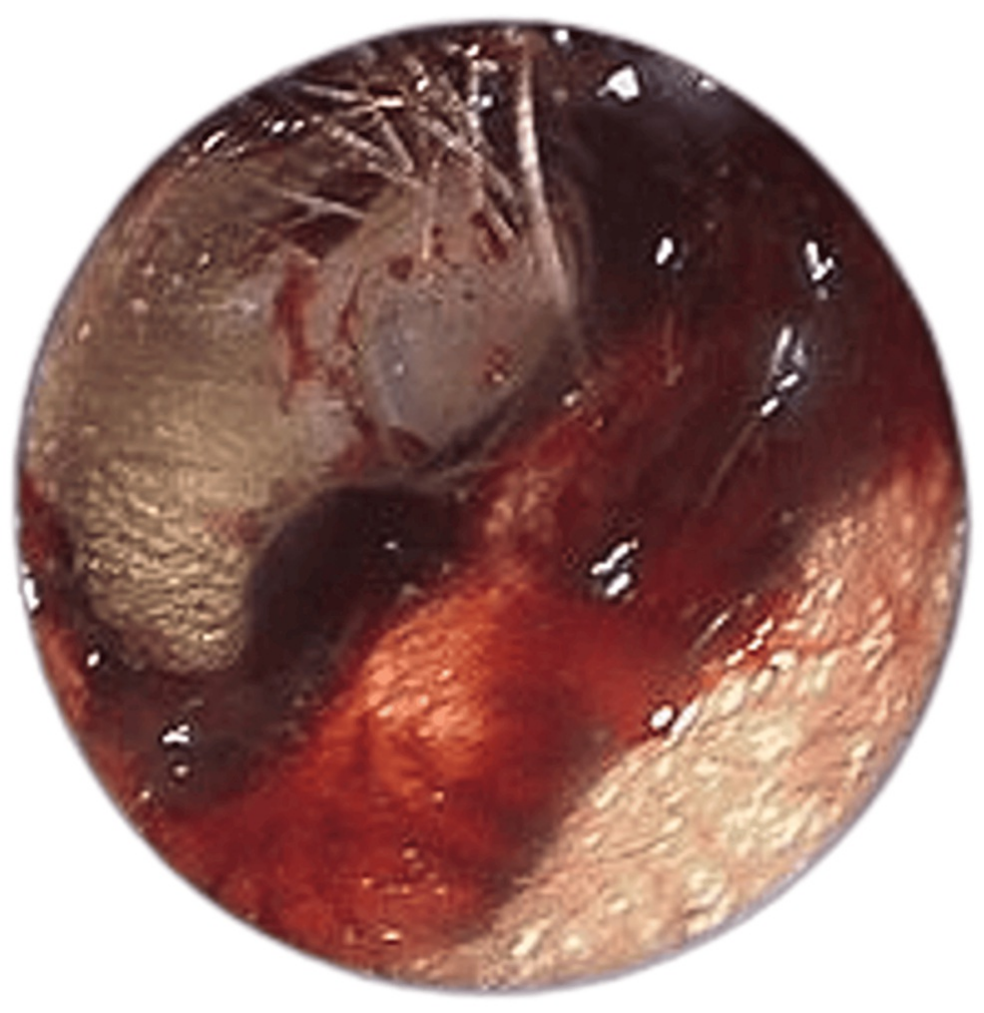
Left external ear Left external ear canal endoscopic examination (pre-ear toileting) showing blood clots in the external auditory canal with intact left tympanic membrane

**Figure 2:**
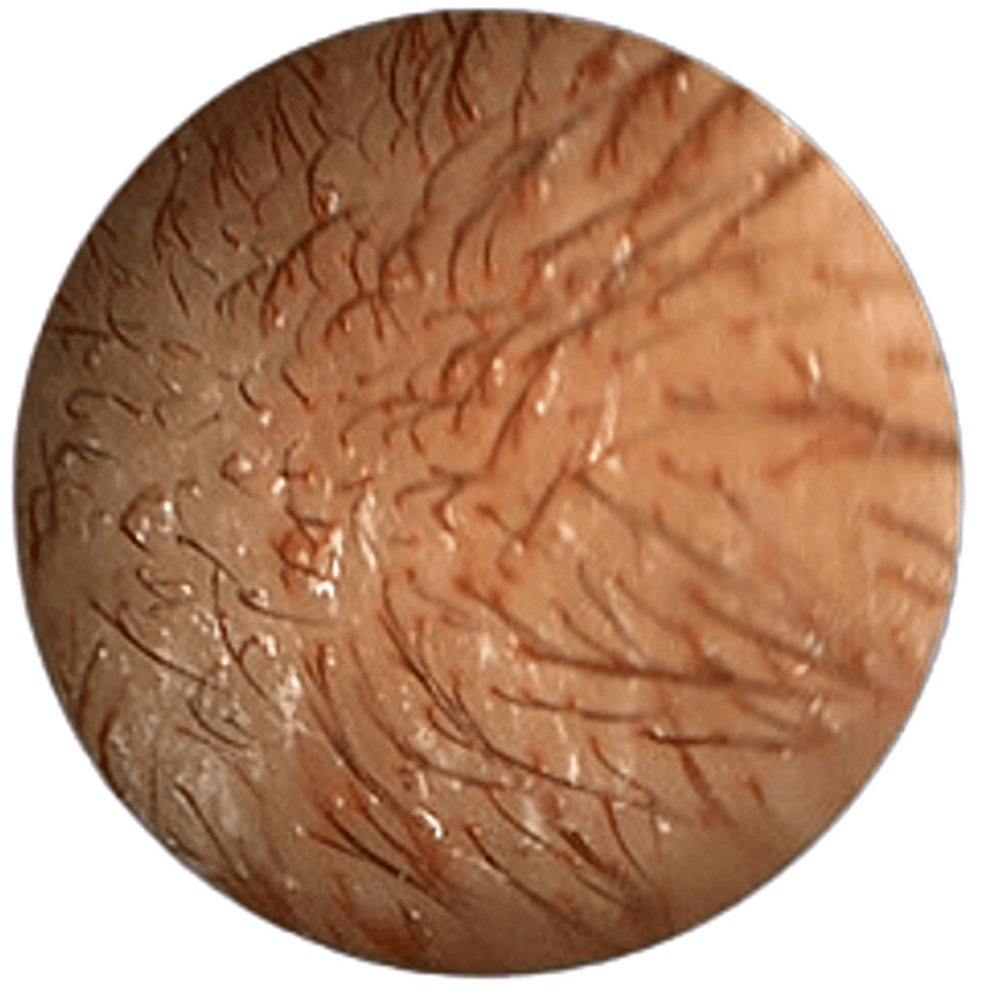
Left external ear canal Left external ear canal endoscopic examination (post-ear toileting) showing blood-stained secretion from the hair roots

A 27-year-old female of Malaysian Indian ethnicity presented with complaints of occasional onset of mild epistaxis and bilateral blood-stained ear discharge for three months. She had no history of trauma or injury to the ears or nasal region. Her blood pressure and pulse rate were within the normal range. Her medical history was unremarkable and she had no significant family history of blood disorders. An anterior rhinoscopy examination revealed dryness over the bilateral Little’s area with no active bleeding. Ear examination demonstrated blood-stained secretion from the hair roots of her skin around the external auditory canal (Figures 1-2).

The patient had no cervical lymphadenopathy, bruises, or hepatosplenomegaly. Our provisional diagnosis was spontaneous ear bleeding. Blood investigations such as serial complete blood count, coagulation profile, and renal profile returned normal. Other tests, including liver function and thyroid function tests, were also normal. She was reviewed by the dermatology team and received regular interval follow-ups subsequently. We started the patient on the β-blocker group tablet propranolol 40 mg daily for two weeks and subsequently followed her up to review her condition. She reported having reduced episodes of bleeding in her ears throughout the β-blocker treatment course.

## Discussion

Hematohidrosis is a rare clinical condition characterized by a self-limiting episode of bloody discharge from the sweat glands due to an unknown cause or external injury. It is often aggravated by psychological stress and is more common in females. The exact pathogenesis of hematohidrosis is still unclear. However, some theories have been proposed, such as vasculitis and sympathetic activation causing engorgement of capillaries, leading to the passage of blood cells through the ducts of sweat glands [5]. In our patient, we observed blood discharge from the hair roots of the bilateral external auditory canal. A normal coagulation profile and liver function test helped exclude other hemorrhagic diseases. Other differentials, such as chromhidrosis (colored sweats), were also excluded as the cause of blood discharge during the visit.

No skin biopsy was taken from the patient, given the inconclusive skin biopsy reported in a previous study. Skin biopsies at the site of bleeding in this previous case report revealed periglandular congested vessels, red blood cells in the follicular lumen and among the collagen fibers, and leakage of blood around dermal capillaries or even normal skin [6]. There are no specific guidelines for managing hematohidrosis. Some studies have reported success in alleviating the symptoms using β-blockers [7,8]. The mechanism of action of β-blockers in treating hematohidrosis is still unclear, although a study has attributed it to the effects of inhibition and angiogenesis [8].

According to one study, a patient achieved complete remission after receiving an atropine transdermal patch. It was applied to the patient's affected areas, and no recurrence was noted during subsequent follow-ups [9]. Psychological evaluation is essential for these patients because hematohidrosis is exacerbated by psychological stress and needs to be addressed with medications [10]. One study has described improvement in symptoms after a therapy that combined oxybutynin and alprazolam in a patient with hematohidrosis aggravated by psychological stress [11].

## Conclusions

Hematohidrosis is a rare condition with an unknown pathogenesis, and no specific guidelines for managing this condition have been established. These patients need frequent follow-ups to monitor the symptoms and complications of blood loss. A proper psychological evaluation needs to be conducted before starting any treatment to get further insights into the condition.
